# Trends and hotspots in tea and Alzheimer's disease research from 2014 to 2023: A bibliometric and visual analysis

**DOI:** 10.1016/j.heliyon.2024.e30063

**Published:** 2024-04-23

**Authors:** Xuefang Meng, Wei Cui, Qian Liang, Bo Zhang, Yingxiu Wei

**Affiliations:** a Department of Pharmacy, Wuming Hospital of Guangxi Medical University, Nanning, China; b Department of Neurology, Wuming Hospital of Guangxi Medical University, Nanning, China; c Department of Scientific Research, Wuming Hospital of Guangxi Medical University, Nanning, China; d Scientific Research Center, Guilin Medical University, Guilin, China

**Keywords:** Bibliometric, Tea, Alzheimer's disease, Polyphenol, Epigallocatechin gallate

## Abstract

**Objectives:**

The positive effects of tea on Alzheimer's disease (AD) have increasingly captured researchers' attention. Nevertheless, the quantitative comprehensive analysis in the relevant literatur is lack. This paper aims to thoroughly examine the current research status and hotspots from 2014 to 2023, providing a valuable reference for subsequent research.

**Methods:**

Documents spanning from 2014 to 2023 were searched from the Web of Science, and the R software, VOSviewer, and Citespace software were used for analysis and visualization.

**Results:**

A total of 374 documents were contained in the study. The rate of article publications exhibited a consistent increase each year from 2014 to 2023. Notably, China emerged as the leading country in terms of published articles, followed by the United States and India. Simultaneously, China is also in a leading position in cooperation with other countries. *Molecules* emerged as the most frequently published journal, while the *Journal of Alzheimer's Disease* secured the top spot in terms of citations. The identified main keywords included oxidative stress, amyloid, epigallocatechin gallate, and green tea polyphenol, among others. These focal areas delved into the antioxidative and anti-amyloid aggregation actions of tea's polyphenolic components. Furthermore, the particularly way in which epigallocatechin gallate delivers neuroprotective outcomes by influencing molecules related to AD represents a focal point of research.

**Conclusion:**

The increasing attention from researchers on the role of tea in ameliorating AD positions it as a hot spot in the development of anti-AD drugs in the development of future. Through our generalized analysis of the current landscape and hotspots regarding tea's application in AD, this study provides an estimable reference for future research endeavors.

## 1 Introduction

Neurodegenerative diseases are on the rise globally, with a significant contribution from the aging population leading to a surge in instances of dementia [1]. According to research statistics, the year 2019 witnessed over 50 million individuals worldwide grappling with dementia, and expected to increase to 152 million by 2050 [2]. Among these, Alzheimer's disease (AD) takes precedence, constituting 60 %–80 % of all reported cases [3]. Its prevalence is mounting at an alarming rate, exerting a profound impact on cognitive function, eroding memory, and potentially hindering an individual's capacity to function autonomously within society. Consequently, this phenomenon has evolved into a substantial public health concern [4,5].

Lifestyle behaviors, including inadequate diet, decreased physical activity, alongside environmental factor, and metabolic risk, are commonly associated with an increased susceptibility to AD [[6], [7], [8]]. Other contributing factors encompass age, gender, smoking, family history of dementia, and genetic predisposition [1,5]. Characteristic manifestations of AD include memory loss and cognitive deterioration [9]. Key neuropathological indicators involve the accumulation of amyloid protein (amyloid-β, Aβ) in cerebral tissue and blood vessels, the formation of neurofibrillary tangles constituted by Tau protein within neurons, and the progressive depletion of synapses [10]. Despite extensive exploration through clinical trials spanning decades, the principal triggers and driving forces behind the disease's progression remain unresolved. Current drug interventions primarily focus on alleviating symptoms [4,11]. Attention regarding AD has shifted towards preemptive measures aimed at risk reduction. Research has elucidated that plant-based foods rich in phenolic compounds and flavonoids exhibit potent antioxidant properties. Especially tea extracts, showcase multifaceted resistance against AD [12].

Tea stands out as a ubiquitous beverage that has garnered substantial attention due to its potential health-enhancing properties [13]. Rich in caffeine and tea polyphenols [14], the advantageous attributes of tea are primarily ascribed to its robust polyphenol content, particularly catechin, renowned for its potent antioxidant capabilities [14]. Regardless of its variety, tea and its derivatives exhibit a spectrum of effects, including antibacterial qualities [15], anti-inflammatory attributes [16], antitumor potential [17], antiviral properties [18], and more. The documented ability of catechin's polyphenols to impede amyloid-beta aggregation and exhibit anti-apoptotic effects suggests a potentially substantial protective role against AD [13,14]. The presence of theanine in tea appears to correlate with improvements in cognitive function [19,20]. Multiple investigations underscore that diverse tea types—ranging from black tea [21] and white tea [22] to green tea [23,24] and Oolong tea [25] — can positively influence mitigating risk factors linked to AD. Nevertheless, the correlation between tea consumption and AD remains an ongoing subject of inquiry, yielding inconclusive findings.

Bibliometrics, an analytical approach with the capacity to delve into extensive publications at both macroscopic and microscopic levels, is a method of scrutinizing books and other communication mediums by using mathematical and statistical methodologies, as articulated by Pritchard. Additionally, Hawkins characterizes it as the quantitative assessment of the bibliographic attributes in literature [26]. The prominence of bibliometrics has notably surged, particularly within medical research. Regrettably, a comprehensive bibliometric analysis of the interplay between tea and AD is absent. To unravel the trajectory of knowledge advancement, pinpoint research focal points, and track research trends concerning the relationship between tea and AD, we utilized R software, VOSviewer, and CiteSpace. These tools enabled us to scrutinize pertinent literature regarding tea and AD, illuminating shifts in research emphases and development trajectories. This analysis aims to uncover evolving research trends in the nexus of AD and tea, potentially identifying nascent research foci that can serve as reference points for forthcoming investigations.

## 2 Materials and methods

### 2.1 Data collection

The data utilized was sourced from WoSCC (Guilin Medical University Purchase Edition) on July 9th, 2023. The search strategy employed the following formula: ((((TS=(tea)) AND TS = ("Alzheimer Dementia*" OR "Alzheimer's Disease" OR "Senile Dementia" OR "Alzheimer Type Dementia" OR "Alzheimer Type Senile Dementia" OR "Primary Senile Degenerative Dementia" OR "Alzheimer Sclerosis" OR "Alzheimer Syndrome" OR "Alzheimer* Diseases" OR "Familial Alzheimer Disease*")) AND PY = (2014–2023)) AND DT = (Article OR Review)) AND LA=(English). The retrieved literatures were saved in plain text format and exported as complete records along with the cited references. Subsequently, superfluous, or redundant literature was expunged from the dataset.

### 2.2 Data analysis

The Bibliometrix package [27] (version 4.0, http://www.bibliometrix.org) within the R software environment (version 3.6.3), alongside VOSviewer [28] (version 1.6.17) and Citespace [29] (version 6.2.4) were used to analyzed. These tools facilitated visual data analysis and the creation of scientific knowledge maps. Two separate authors independently undertook the tasks of data extraction and analysis to guarantee accuracy and reliability of the data.

The visualizations of national collaborator networks, co-citation analysis of sources, and co-occurrence of keywords were generated by VOSviewer. The criteria employed for co-authorship network analysis were as follows: one country should have a minimum of four documents. For co-citation source analysis, a source required a minimum of 15 citations. Additionally, for co-occurrence keyword analysis, a keyword needed a minimum of 5 occurrences, excluding keywords like "tea," "AD," and "Alzheimer's disease" from consideration. Furthermore, Impact Factors (IF) for journals were sourced from the 2022 Journal Citation Reports (JCR).

## 3 Results

### 3.1 Overview of selected studies on tea in Alzheimer's disease

**Fig. 1:**
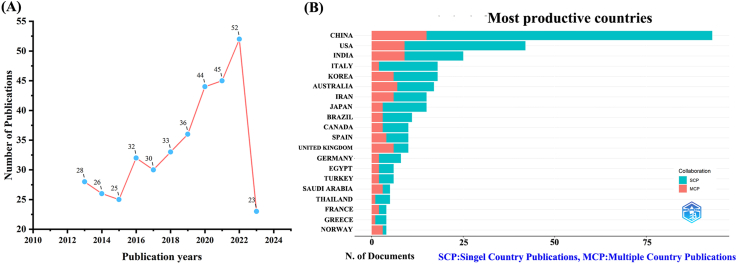
Trends in annual publication outputs in the field of tea in AD from 2014 to 2023. (A) Trends of annual publication outputs. (B) Showcases the distribution of countries and collaborative efforts among corresponding authors.

A total of 374 unique literatures were collected from WoSCC, ensuring the removal of duplicates. Over the period from 2014 to 2023, the number of documents related to tea's involvement in AD demonstrates a consistent upward trajectory, as depicted in Fig. 1A. This trend signifies a growing interest among researchers in exploring the interplay between tea and AD.

**Fig. 2:**
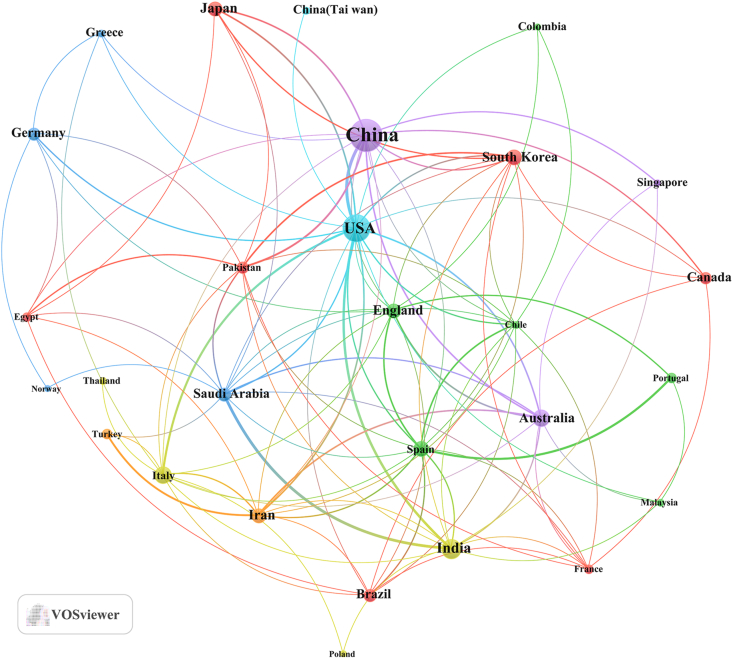
The map of countries and institutions in the field of tea in AD from 2014 to 2023.

Regarding the corresponding authors' countries, an examination revealed that China (n = 93) emerged as the most publish contributor, followed by the USA (n = 42), India (n = 25), Italy (n = 18), and Korea (n = 18). Furthermore, only 11.10 % of Italy's publications and 16.10 % of China's publications were attributed to multiple-country collaborations (MCPs), significantly lower than the rates of 33.30 % in Korea and 36.00 % in India, as depicted in Fig. 1B and summarized in Table 1. Interestingly, despite China's dominance in document output, the USA exhibited a more extensive network of collaborative ties with other nations, serving as a focal point for cooperative endeavors, as shown in Fig. 2.

**Table 1:** Most relevant countries by corresponding authors of tea in AD.

Country	Articles	SCP	MCP	Freq	MCP_Ratio
China	93	78	15	0.249	0.161
USA	42	33	9	0.112	0.214
India	25	16	9	0.067	0.36
Italy	18	16	2	0.048	0.111
Korea	18	12	6	0.048	0.333
Australia	17	10	7	0.045	0.412
Iran	15	9	6	0.04	0.4
Japan	15	12	3	0.04	0.2
Brazil	11	8	3	0.029	0.273
Canada	10	7	3	0.027	0.3
Spain	10	6	4	0.027	0.4
United Kingdom	10	4	6	0.027	0.6
Germany	8	6	2	0.021	0.25
Egypt	6	4	2	0.016	0.333
Turkey	6	4	2	0.016	0.333
Saudi Arabia	5	2	3	0.013	0.6
Thailand	5	4	1	0.013	0.2
France	4	2	2	0.011	0.5
Greece	4	3	1	0.011	0.25
Norway	4	1	3	0.011	0.75

Note: MCP: Multiple country publication; SCP: Single country publication.

### 3.2 Journal analysis and visualization

To analyze the journals contributing the most publications and citations within the context of tea in AD, we employed the Bibliometrix package within R software. Visualizations were generated using the ggplot2 package. Additionally, VOSviewer was utilized for co-cited journal analysis.

**Fig. 3:**
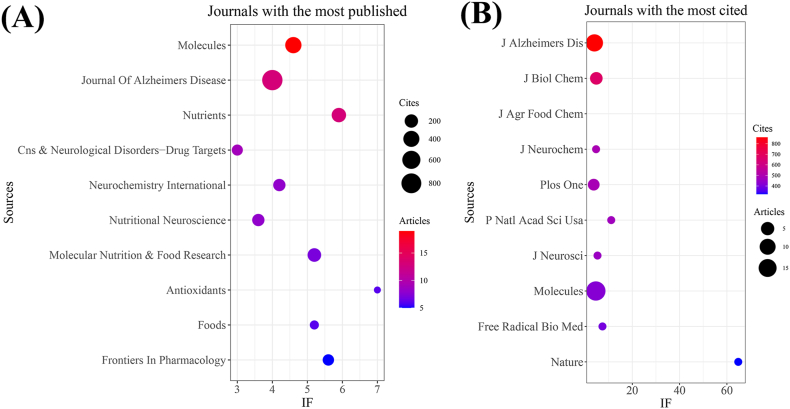
The journal with the largest number of articles published and the journal with the largest number of citations. (A) The journal with the highest count of published documents. (B) The journals with the highest count of citations.

**Fig. 4:**
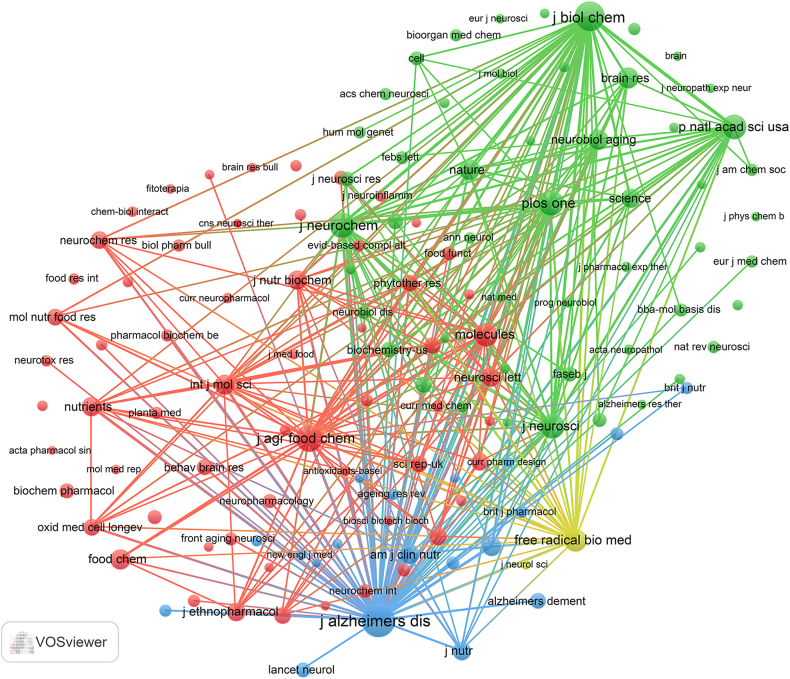
Co-cited journals involved in tea in AD.

From our investigation, we discovered a total of 374 documents distributed across 193 academic journals (refer to Annexes 1 for further details). As presented in Table 2 and depicted in Fig. 3A, *Molecules* (n = 19, IF = 4.6) emerged as the journal with the largest number of published documents, followed by the *Journal of Alzheimer's Disease* (n = 13, IF = 4), *Nutrients* (n = 13, IF = 5.9), *CNS & Neurological Disorders-Drug Targets* (n = 9, IF = 3), and *Neurochemistry International* (n = 8, IF = 4.2). Table 3 and Fig. 3B highlight the most frequently cited journals, including the *Journal of Alzheimer's Disease* (n = 860, IF = 4), *Journal of Biological Chemistry* (n = 674, IF = 4.8), *Journal of Agricultural and Food Chemistry* (n = 569, IF = 4.8), *Journal of Neurochemistry* (n = 492, IF = 4.7), and *Plos One* (n = 488, IF = 3.7). Notably, the co-cited journals map in Fig. 4 reveals that the Journal of Alzheimer's Disease, Molecules, and Journal of Biological Chemistry serve as central hubs of collaboration. These findings collectively emphasize the importance of the *Journal of Alzheimer's Disease* and *Molecules* as influential journals in the domain of tea's association with AD.

**Table 2:** Top 10 journals with the most published.

Sources	Documents	IF (2022)	Cites	JCR	JCR ranking
Molecules	19	4.6	419	Q2	97/285
Journal of Alzheimer's Disease	13	4	860	Q2	105/272
Nutrients	13	5.9	269	Q1	17/88
CNS & Neurological Disorders-Drug Targets	9	3	83	Q3	163/272
Neurochemistry International	8	4.2	139	Q2	111/285
Nutritional Neuroscience	8	3.6	139	Q2	119/272
Molecular Nutrition & Food Research	7	5.2	214	Q1	34/142
Antioxidants	6	7	1	Q1	46/285
Foods	6	5.2	23	Q1	34/142
Frontiers In Pharmacology	5	5.6	96	Q1	45/277

**Table 3:** Top 10 journals with the most cited.

Sources	Cites	IF (2022)	Documents	JCR	JCR ranking
Journal of Alzheimer's Disease	860	4	13	Q2	105/272
Journal of Biological Chemistry	674	4.8	4	Q2	88/285
Journal of Agricultural and Food Chemistry	569	4.8	5	Q1	6/58
Journal of Neurochemistry	492	4.7	1	Q2	92/285
Plos One	488	3.7	3	Q2	26/73
P Natl Acad Sci Usa	471	11.1	1	Q1	8/73
Journal of Neuroscience	458	5.3	0	Q1	62/272
Molecules	419	4.6	19	Q2	97/285
Free Radical Biology and Medicine	396	7.4	1	Q1	43/285
Nature	323	64.8	1	Q1	1/73

### 3.3 Analysis of cited-references

**Fig. 5:**
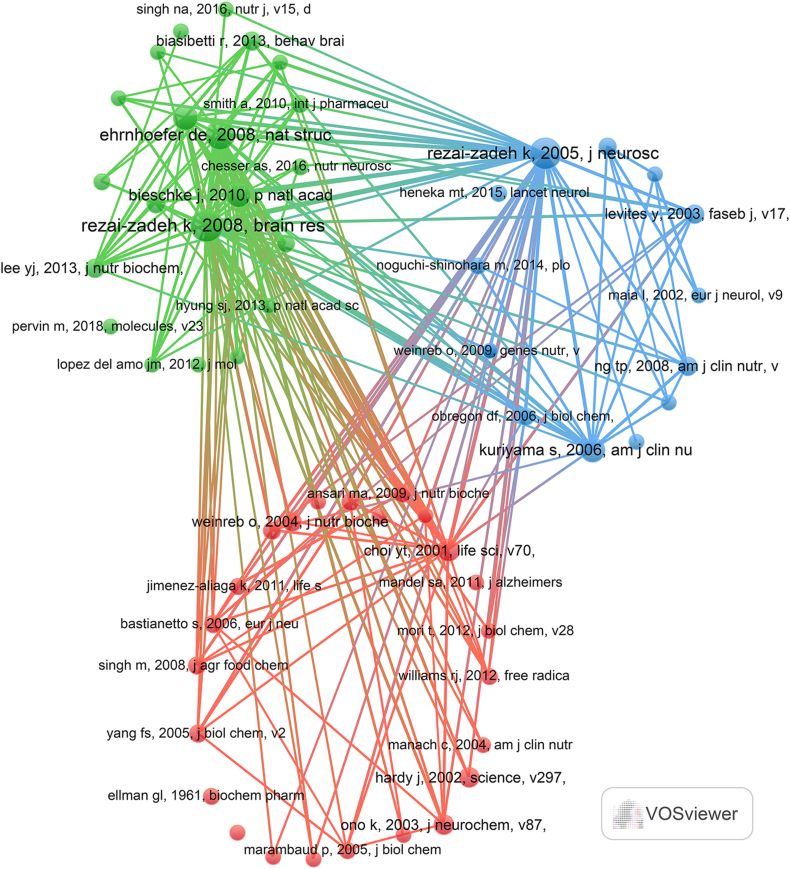
Co-citation involved in tea in AD.

VOSviewer was utilized for co-citation analysis was related to the tea in AD (Fig. 5, Table 4). We discovered that these co-citations predominantly center on the mechanism of epigallocatechin gallate (EGCG) in treating AD, investigations into the correlation between consumption of green tea and cognitive function, and the therapeutic impacts of other active drug ingredients on AD. Notably, the research emphasis is on the mechanism of EGCG in AD treatment, encompassing its influence on protein aggregation and neuroprotection, as well as its impact on neuroinflammation and oxidative stress. Consequently, the mechanism by which EGCG contributes to the treatment of AD may represent a significant area of focus within this field.

**Table 4:** The top 20 co-citation related to the tea in AD.

Paper	DOI	Total co-citations
Rezai-Zadeh K, 2008, Brain Res,	10.1016/j.brainres.2008.02.107	71
Rezai-Zadeh K, 2005, J Neurosci,	10.1523/jneurosci.1521–05.2005	68
Ehrnhoefer De, 2008, Nat Struct Mol Biol,	10.1038/nsmb.1437	55
Bieschke J, 2010, P Natl Acad Sci Usa,	10.1073/pnas.0910723107	42
Kuriyama S, 2006, Am J Clin Nutr,	10.1093/ajcn/83.2.355	41
Lee Jw, 2009, J Nutr,	10.3945/jn.109.109785	40
Choi Yt, 2001, Life Sci,	10.1016/s0024-3205(01)01438-2	33
Hardy J, 2002, Science,	10.1126/science.1072994	27
Ono K, 2003, J Neurochem,	10.1046/j.1471–4159.2003.01976.x	26
Dragice n, 2011, J alzheimers dis	10.3233/jad-2011-101629	25
Weinreb O, 2004, J Nutr Biochem,	10.1016/j.jnutbio.2004.05.002	25
Lee Yj, 2013, J Nutr Biochem,	10.1016/j.jnutbio.2012.06.011	24
Levites y, 2003, Faseb j	10.1096/fj.02-0881fje	24
Ng Tp, 2008, Am J Clin Nutr,	10.1093/ajcn/88.1.224	24
Biasibetti R, 2013, Beha	10.1016/j.bbr.2012.08.039	23
Ansari Ma, 2009, J Nutr Biochem,	10.1016/j.jnutbio.2008.03.002	22
Bastianetto S, 2006, Eur J Neurosci,	10.1111/j.1460–9568.2005.04532.x	22
Obregon Df, 2006, J Biol Chem,	10.1074/jbc.m600617200	22
Haque Am, 2008, J Nutr Biochem,	10.1016/j.jnutbio.2007.08.008	21
Yang Fs, 2005, J Biol Chem,	10.1074/jbc.m404751200	21

**Fig. 6:**
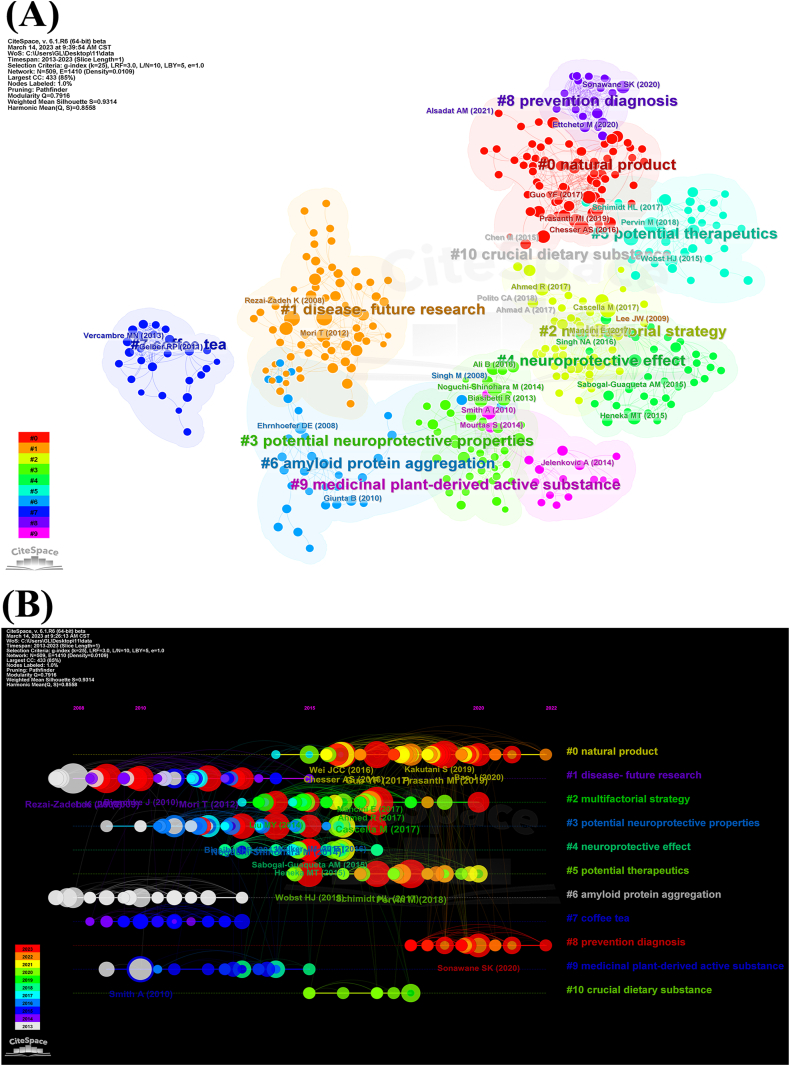
Reference cluster analysis and temporal mapping on tea in AD. (A) Reference cluster analysis on tea in AD. (B) Temporal mapping of reference clusters on tea in AD.

At the same time, CiteSpace was used for reference cluster analysis. As illustrated in Fig. 6A, citations were segmented into 10 clusters (individual citations were not depicted in the figure): #0 natural product; #1 disease-future research; #2 multifactorial strategy; #3 potential neuroprotective properties; #4 neuroprotective effect; #5 potential therapeutics; #6 amyloid protein aggregation; #7 coffee tea; #8 prevention diagnosis; #9 medicinal plant-derived active substance; #10 crucial dietary substance. According to the degree value of each point in the cluster, we place the top ten citations in each cluster in **Annex 2**. Through analysis, it was found that the ten clusters could be summarized into two research directions: 1. Natural active ingredients for treating AD (#0, #7, #9, #10), including EGCG, polyphenol, flavonoids and so on; 2. Strategy and mechanism of diagnosis and treatment of AD (#1–6, #8), including antioxidant, protective effect in primary neurons against Abeta, antiamyloidogenic and fibril-disaggregating and so on. Interestingly, of all the top ten citations in the ten clusters, 16 % were related to EGCG. This result was in accordance with the results of co-citation analysis. To further elucidate the research frontiers and trends within this field, a temporal mapping of these clusters was conducted, with bold timelines indicating periods where the clustered topics emerged as hotspots (Fig. 6B). This visualization aids in understanding the temporal dynamics of topics within the field and facilitates exploration of its evolutionary trajectory.

In addition, the Bibliometrix package within the R software was used to determine the top 20 most cited references in the context of tea's association with AD (Table 5). These references accumulated over 110 citations each and were dispersed across 19 different journals, indicating a lack of dominance by any specific journal.

**Table 5:** The top 20 cited references related to the tea in AD.

Paper	DOI	Total Citations	TC per Year
Xing Lj, 2019, J Agr Food Chem	10.1021/acs.jafc.8b06146	253	50.6
Batiha Ge, 2020, Foods	10.3390/foods9030374	238	59.5
Bhullar Ks, 2013, Oxid Med Cell Longev	10.1155/2013/891748	236	21.45
He Xr, 2014, J Ethnopharmacol	10.1016/j.jep.2013.11.023	207	20.7
Hyung Sj, 2013, P Natl Acad Sci Usa	10.1073/pnas.1220326110	182	16.55
Cheng B, 2013, Bba-Gen Subjects	10.1016/j.bbagen.2013.06.029	171	15.55
Ayaz M, 2019, Front Aging Neurosci	10.3389/fnagi.2019.00155	167	33.4
Singh Na, 2016, Nutr J	10.1186/s12937-016-0179-4	165	20.63
Prasanth Mi, 2019, Nutrients	10.3390/nu11020474	161	32.2
Zaplatic E, 2019, Life Sci	10.1016/j.lfs.2019.03.055	156	31.2
Cano A, 2019, J Control Release	10.1016/j.jconrel.2019.03.010	144	28.8
Hu N, 2013, Biomed Res Int	10.1155/2013/524820	138	12.55
Wobst Hj, 2015, Febs Lett	10.1016/j.febslet.2014.11.026	137	15.22
Lee Yj, 2013, J Nutr Biochem	10.1016/j.jnutbio.2012.06.011	130	11.82
Panza F, 2015, J Nutr Health Aging	10.1007/s12603-014-0563-8	129	14.33
Pervin M, 2018, Molecules	10.3390/molecules23061297	125	20.83
Simunkova M, 2019, Arch Toxicol	10.1007/s00204-019-02538-y	125	25
Shal B, 2018, Front Pharmacol	10.3389/fphar.2018.00548	119	19.83
Rahman Mm, 2022, Molecules	10.3390/molecules27010233	119	59.5
Di Marco Ly, 2014, J Alzheimers Dis	10.3233/JAD-132225	111	11.1

Note: “TC per Year” is an abbreviation for “Total Citations per Year”.

The top three cited references were: "Recent advances in the understanding of the health benefits and molecular mechanisms associated with green tea polyphenols," "The pharmacological activity, biochemical properties, and pharmacokinetics of the major natural polyphenolic flavonoid: quercetin," and "Polyphenols: multipotent therapeutic agents in neurodegenerative diseases." Through integrated analysis, we observed that these references predominantly revolve around four key themes: (1) The health benefits and molecular mechanisms associated with green tea polyphenols; (2) Pharmacological attributes of green tea polyphenols, covering their biochemical traits and pharmacokinetics; (3) The roles of polyphenols in addressing neurodegenerative diseases; (4) Strategies for curtailing amyloidogenesis and conferring neuroprotection. These focal areas collectively highlight the pivotal aspects that underpinning the synergy between tea and AD research.

**Fig. 7:**
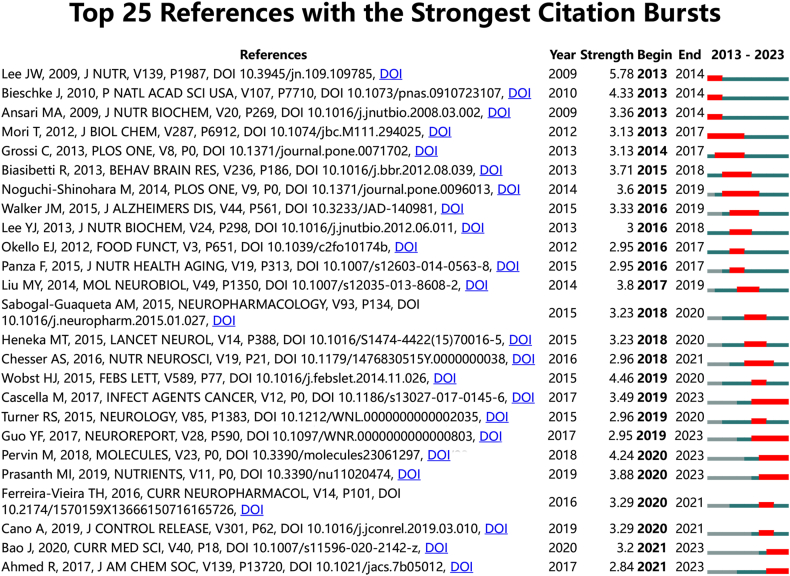
Top 25 References with the strongest citation bursts on tea in AD.

We employed CiteSpace to determine the top 25 most important citation bursts for tea in AD (Fig. 7), and the titles of these citations are provided in **Annex 3** along with their corresponding DOIs. Notably, the top three citations with the most intense citation bursts were: (1) “Green tea (−)-epigallocatechin-3-gallate inhibits beta-amyloid-induced cognitive dysfunction through modification of secretase activity via inhibition of ERK and NF-kappaB pathways in mice (strength: 5.78)”. (2) “The green tea polyphenol (−)-epigallocatechin gallate prevents the aggregation of tau protein into toxic oligomers at substoichiometric ratios (strength: 4.46)”. (3) “EGCG remodels mature alpha-synuclein and amyloid-beta fibrils and reduces cellular toxicity (strength: 4.33)”. In addition, titles of the three most leading-edge citation bursts were: (1) Dual-drug loaded nanoparticles of Epigallocatechin-3-gallate (EGCG)/Ascorbic acid enhance therapeutic efficacy of EGCG in a APPswe/PS1dE9 Alzheimer's disease mice model. (2) Epigallocatechin-3-gallate Alleviates Cognitive Deficits in APP/PS1 Mice”. (3) “Molecular Mechanism for the (−)-Epigallocatechin Gallate-Induced Toxic to Nontoxic Remodeling of A beta Oligomers. After that, we conducted an inductive analysis of 25 citations and found that they mainly contain three themes: (1) Mechanism of action of tea compounds in the treatment of AD; (2) Natural Compounds and neuroprotection and inflammation inhibition; (3. The intake and cognitive function of tea and coffee. Overall, through the most cited references and reference burst analysis, we can find that "active ingredients in tea and neuroprotective mechanism" may be the hot spots in the field of tea in AD. In summary, the most cited references and reference burst analysis suggest that the "active ingredients in tea and neuroprotective mechanisms" may be the current hotspots in the field between tea and AD.

### 3.4 Keyword clusters and evolution of themes

Keyword clusters play a crucial role in quickly understanding the research hotspots and trends within a specific field. In our analysis, 2307 keywords were identified through VOSviewer, with Table 6 showcasing the top 20 keywords that appeared more than 20 times. The most frequently appearing keyword was “polyphenol” (n = 144), followed by “oxidative stress” (n = 133), “amyloid” (n = 101), “epigallocatechin gallate” (n = 92), “antioxidant” (n = 63), “cognitive impairment” (n = 61), and “dementia” (n = 45).

**Table 6:** The top 20 keywords related to tea in AD.

Rank	Keywords	Count
1	polyphenol	144
2	oxidative stress	133
3	amyloid	101
4	epigallocatechin gallate	92
5	antioxidant	63
6	cognitive impairment	61
7	dementia	45
8	in-vitro	37
9	brain	34
10	neuroprotection	34
11	neurodegenerative diseases	30
12	nf-kappa-b	29
13	flavonoids	28
14	a-beta	27
15	acetylcholinesterase	25
16	mouse model	24
17	inflammation	23
18	parkinsons-disease	23
19	neurodegeneration	22
20	protein	21

**Fig. 8:**
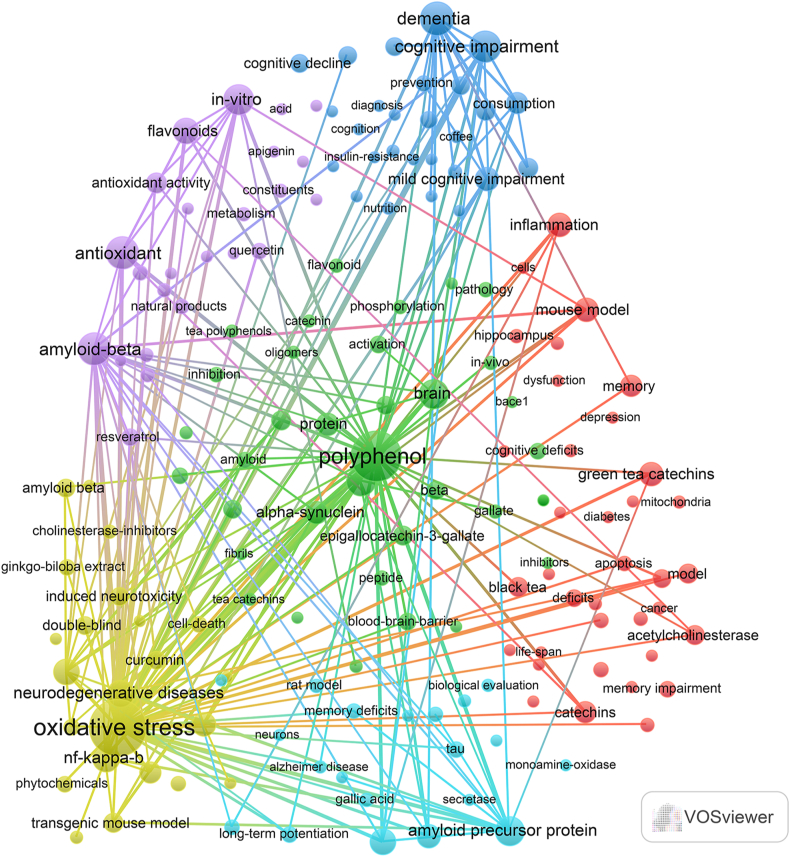
Keywords co-occurrence map of publications on tea in AD.

Through cluster analysis, we observe six different colored clusters in Fig. 8. (1) In "Clustering of neuroprotective effects of tea extracts on Alzheimer's disease patients and their mechanisms" (red dots), there are 37 keywords, including green tea catechins, acetylcholinesterase, memory impairment, apoptosis, lipid peroxidation, and so on. (2) In the "Clustering of the effects of tea extracts on pathomechanisms related to Alzheimer's disease" (green dots), there are 37 keywords, including epigallocatechin gallate(EGCG), polyphenol, protein, brain, phosphorylation, etc. (3) The cluster "Effects and mechanisms of tea in preventing cognitive decline" (dark blue dots) has 27 keywords, including cognitive impairment, prevention, caffeine, amyloid-β levels, etc. (4) In the cluster "Pathogenesis of neurodegenerative diseases and drug research" (yellow dot), there are 25 keywords, including oxidative stress, neurodegenerative disease, neuroinflammation, amyloid beta peptide, curcumin, and so on. (5) In the cluster "Phytochemicals and neuroprotection: potential therapeutic agents for neurological diseases" (purple dot), there are 24 keywords, including antioxidant, neuroprotective, flavonoids, phenolic compounds, and so on. (6) In the cluster "Effect of tea on Alzheimer's disease and its related pathophysiological processes" (light blue dots), there are 20 keywords, including amyloid precursor protein, neurodegeneration, long-term potentiation, neuroprotection, and gallic acid. (refer to **Annex 4**).

**Fig. 9:**
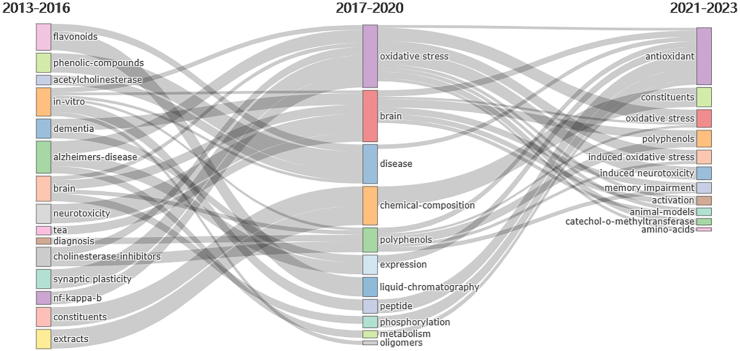
Trend topics on tea in AD research.

In addition, to predict the future directions of this field, we used the bibliometrix package of R software to generate a thematic evolution (Fig. 9). Analysis reveals that from 2013 to 2016, research in this domain predominantly concentrated on the active constituents of tea. The period from 2017 to 2020 saw a shift in focus towards the antioxidant effects, marking it as the primary area of interest, while the emphasis on tea's active components saw a slight decline. Between 2021 and 2023, antioxidant effects continued to be a focal point of research, with polyphenols, as active constituents of tea for the amelioration of AD, garnering increasing attention. This aligns with the outcomes derived from the temporal mapping of reference clusters. As a result, future research in this domain is expected to focus on the impact of tea's active components, especially polyphenols, and their antioxidant capabilities in fighting Alzheimer's disease.

In summary, our comprehensive analysis, incorporating citation analysis, reference cluster analysis, keyword frequency analysis, keyword clustering, and thematic evolution, has unveiled potential research hotspots in the field between tea and AD. These focal points revolve around the antioxidative and anti-amyloid aggregation mechanisms inherent in the polyphenolic components of tea. In addition, EGCG is deemed an effective component in AD treatment, and elucidating the specific mechanism behind EGCG's neuroprotective effects related to AD constitutes a prominent research focus in this field.

## 4 Discussion

### 4.1 General information

Within the scope of this study, we compiled a comprehensive set of 374 documents, spanning the period from 2014 to 2023. The discerned pattern reveals a consistent upward trajectory in the number of documents exploring the interaction between tea and AD, indicating a progressive escalation over the years. This unmistakable trend underscores the growing appeal and prominence of tea in AD research within the academic community. In the domain of tea in AD research, China was outstanding as the leading contributor, producing the largest volume of scholarly papers. This trend aligns with the historical roots of tea in China, justifying the considerable attention it receives from Chinese researchers.

A comprehensive collection of 374 documents was distributed across 193 journals. Leading contributors included esteemed journals such as Molecules, Journal of Alzheimer's Disease, Nutrients, CNS & Neurological Disorders-Drug Targets, and Neurochemistry International. Notably, the Journal of Alzheimer's Disease stood out as a significant focal point, boasting both a substantial count of published articles and a considerable volume of citations. This prominence positions the Journal of Alzheimer's Disease as a pivotal publication within the realm of tea's intersection with AD research, solidifying its role as a primary conduit for disseminating research findings in this domain. Additionally, our analysis reveals that the top 20 most cited references exploring the link between consumption of tea and AD have garnered over 110 citations each, spread across 19 distinct journals. This distribution suggests a diverse interest across the field without a predominant focus on any single journal.

### 4.2 Hotspots and development trends

Through an in-depth analysis of the co-citation, reference cluster analysis, most cited references, reference bursts, keyword clusters, and thematic evolution, we have successfully determined three prominent research hotspots and discernible development trends in the intersection of tea and AD.

Firstly, a pivotal mechanism revolves around the antioxidative properties of polyphenolic constituents found in tea in the context of AD. The antioxidative mechanism emerges as one of the pathways through which these polyphenols may exert an influence on AD. Oxidative stress, characterized by an imbalance between reactive oxygen species (ROS) production and the body's antioxidative defenses, plays a recognized and substantial role in the development and progression of AD [30]. This oxidative stress leads to cellular damage, including harm to neurons and neuronal membranes, thereby contributing to the observed cognitive decline in AD [31]. Polyphenols encompass a diverse group of compounds that are abundant in tea, particularly in green tea [32]. Notably, compounds like EGCG in green tea showcase robust antioxidative properties [33]. Functioning as effective free radical scavengers, they neutralize ROS and reactive nitrogen species (RNS), which have the potential to cause cellular damage [34]. Furthermore, the antioxidative effects attributed to polyphenols hold promise for contributing to neuroprotection. By mitigating oxidative stress and preventing harm from ROS and RNS, polyphenols may actively support the maintaining of both neuronal function and integrity [35]. In doing so, they potentially provide a means to slow down cognitive decline in individuals affected by AD.

Secondly, a crucial mechanism involves the inhibition of amyloid aggregation in AD by polyphenolic components present in tea. A hallmark feature of AD is the abnormal accumulation of β-amyloid, leading to the formation of amyloid plaques [36]. These plaques disrupt normal neuronal function, ultimately contributing to cognitive decline. Among tea's polyphenolic constituents, catechins, in particular, are believed to possess the potential to impede β-amyloid aggregation [37]. Polyphenols can intervene in the aggregation process by interacting directly with β-amyloid molecules [38]. For example, catechins demonstrate the ability to establish hydrogen bonds and engage in interactions between tea polyphenols and the hydrophilic amino acids on the surface of β-amyloid. This interaction serves to hinder or slow down the aggregation process [39]. Additionally, polyphenols may exert their aggregation-inhibiting influence by enhancing the extracellular elimination of amyloid [40]. This can be achieved by reinforcing processes such as phagocytosis carried out by macrophages or other analogous clearance mechanisms.

Thirdly, the specific mechanism by which EGCG exerts neuroprotective effects by modulating molecular associated with AD is one of the research hotspots in this field. Researches have demonstrated that the green tea polyphenol compound EGCG preserves mitochondrial energy, limits inflammation, and reduces neuronal damage in the brain. Additionally, it promotes synapse growth, thus exhibiting neurorescue activity [41]. Furthermore, investigations suggest that bioactive components of tea, including EGCG, could serve as drug candidates for AD treatment, potentially in combination with nanotechnology, offering a safe and effective therapeutic strategy [42]. EGCG has also been found to alleviate neuroinflammation by reducing microglial cell activation, thereby slowing immune senescence. Collectively, these studies highlight EGCG as a promising approach to combat neurodegenerative diseases like AD [43]. In terms of mechanism, research indicates that the pathological feature of AD involves extracellular plaques composed of fibrillar Aβ peptides and intracellular neurofibrillary tangles composed of hyperphosphorylated tau proteins. EGCG exerts neuroprotective effects by regulating amyloid precursor protein (APP) processing to inhibit β-amyloid peptide (Aβ) deposition, attenuating Aβ-induced oxidative stress, and reducing Aβ-induced neuroinflammatory responses [44]. Additionally, EGCG significantly inhibits the aggregation of the protein sAPPβ and reverses Ca^2+^-induced neuroinflammatory responses. It also modulates Ca^2+^ endocytosis, inhibits the activation of the transcription factor NF-kappa B, and reduces the secretion of pro-inflammatory IL-6 in astrocyte-like cells, thus inhibiting inflammation and exerting neuroprotective effects [45]. However, despite numerous studies investigating the specific mechanisms of EGCG's neuroprotective effects on AD molecular, its pharmacological mechanisms remain largely unexplored, and strategies for its use in AD treatment require further exploration [46]. Therefore, it is reasonable to assume that the specific mechanism by which EGCG exerts a neuroprotective effect by modulating the molecular associated with AD will be one of the research hotspots in this field.

It's crucial to note that while laboratory and preclinical studies offer compelling evidence for the antioxidative properties of tea polyphenols and their potential benefits in AD, translating these findings into effective treatments for human patients is a complex challenge. Clinical trials are indispensable to acquire a deeper understanding of the efficacy, optimal dosage, and long-term effects of tea consumption or polyphenol supplements in the context of AD.

### 4.3 Limitations

It is essential to acknowledge that, while our study holds the potential to facilitate a rapid understanding of the field's research focal points and cutting-edge trends, it does have inherent limitations. Primarily, the data sources used for our research were exclusively drawn from the WoSCC, introducing the possibility of excluding pertinent literature from other databases. Nevertheless, it is crucial to emphasize that the WoS database holds a preeminent position as a digital literature repository, highly regarded by scholars and universally deemed as the most suitable platform for conducting bibliometric analyses [47,48]. To bolster the robustness of our study, we extended our search to encompass the PubMed and Scopus databases. The findings from these two additional databases substantiate that our study encapsulates almost the entirety of the available data. This validation reinforces the reliability of our research.

A secondary limitation pertains to our inclusion criteria, which exclusively considered English-language studies. This choice potentially introduces a bias in our findings by overlooking non-English literature. Furthermore, it is noteworthy that we chose not to undertake an analysis of issuing institutions. This decision is rooted in our observation that research institutions within this field exhibit limited collaboration, rendering the analytical value of such an investigation relatively modest.

In short, notwithstanding the acknowledged constraints, our study provides a comprehensive summary of the current landscape, key focal points, and investigative trajectories within this field.

## 5 Conclusion

This study elucidates the primary research hotspots and frontiers between tea and AD. The following provides a brief overview of the key information and current areas of intensive research within this domain:

a. The investigation into tea's impact on AD has garnered global interest, attracting notable engagement from scholars worldwide, particularly from nations such as China, the USA, India, Italy, and Korea. This research landscape is characterized by robust international collaborations.
b. Molecules and the Journal of Alzheimer's Disease emerge as the primary journals consistently publishing materials related to tea in AD. Notably, the Journal of Alzheimer's Disease holds the distinction of being the most referenced publication, underscoring its pivotal role as a seminal journal in the study domain focused on the impact between tea and AD.
c. Tea polyphenols are the key active ingredients in the treatment of AD.
d. The antioxidant and anti-amyloid aggregation effects of tea polyphenols are key research focal points in the treatment of AD.
e. The specific mechanism by which EGCG exerts neuroprotective effects by modulating molecular associated with AD is the research hotspots in this field.

In conclusion, our research provides invaluable opinions into the emerging trends and central themes within the realm of tea in AD. These findings aim to assist scholars in achieving a comprehensive understanding of this specialized field, facilitating the investigation of new way for future investigations. By delineating the current research landscape and prospective focal points, our study provides important information for researchers to master this field more adeptly. This, in turn, empowers them to embark on innovative trajectories in their inquiries with heightened efficacy and purpose.

## Data availability statement

The raw data supporting the conclusion of this article will be made available by the authors, without undue reservation.

## Funding

This research was supported by Youth Science Foundation of Guangxi Medical University（No.GXMUYSF202355).

## CRediT authorship contribution statement

**Xuefang Meng:** Writing – original draft. **Wei Cui:** Data curation. **Qian Liang:** Visualization. **Bo Zhang:** Visualization. **Yingxiu Wei:** Writing – review & editing, Writing – original draft.

## Declaration of competing interest

The authors declare that they have no known competing financial interests or personal relationships that could have appeared to influence the work reported in this paper.
